# Moving Towards a South African NHI System of Excellence: Recommendations Based on the Insider Perspectives of CHWs as Key Role-Players

**DOI:** 10.3390/ijerph22050807

**Published:** 2025-05-21

**Authors:** Corlia Janse van Vuuren, Zanette Lowe, Karen Bodenstein

**Affiliations:** 1 School of Health & Rehabilitation Sciences, University of the Free State (UFS), Bloemfontein 9301, South Africa; 2 Department of Physiotherapy, University of the Free State (UFS), Bloemfontein 9301, South Africa; zanette04@gmail.com (Z.L.); bodensteink@ufs.ac.za (K.B.)

**Keywords:** community health workers (CHWs), health promotion, re-engineered primary health care, National Health Insurance (NHI), South African health care system

## Abstract

Aligned with the worldwide shift towards promotional and preventative health care, the South African government has introduced a re-engineered primary health care plan, facilitated through a National Health Insurance (NHI) platform. Community health workers (CHWs) are key role-players in most universal health care systems. This article shares insider perspectives from 31 CHWs in one of the South African NHI pilot districts. Based on their perspectives, the authors share recommendations to strengthen the NHI plan. Recommendations comprise of the inclusion of a dedicated CHW team leader and reporting nurse, ongoing CHW education and training with an accompanying portfolio of evidence, and awareness campaigns on the role of CHWs within the South African re-engineered primary health care plan and NHI platform.

## 1. Introduction

The goal of any health care system is to improve and promote the health of the people by providing access to quality services that are efficient and effective. Worldwide, many countries recognise the importance of a preventative and promotional approach rather than a curative approach, especially at primary health care (PHC) levels, in alignment with the World Health Organisation directives [1].

Faced by disproportionate access and quality of health care in South Africa (SA), the South African government proposed a new health plan to re-engineer PHC within the country to reach a larger proportion of the population with quality health care services. This initiative is facilitated through the introduction of a National Health Insurance (NHI) plan developed to co-currently restructure the entire concept of health insurance and patient care within SA [2]. The re-engineered PHC plan will, besides widening access, attempt to shift the focus from hospitalisation to the management of health conditions at primary and community care level [2].

The human resource crisis in the health care sector, faced by many middle- and low-income countries, especially in Africa, remains the main challenge to providing extended health services to the community. Mupara et al. describe the inclusion of community health workers (CHWs) in such health plans as a possible solution [3]. CHWs are expected to render services that are aimed at addressing the specific needs of the community, which create a sense of ownership and responsibility within the community [4], but are, at the same time, more cost-effective for the government. The overall coverage of health services is therefore expected to improve, as these workers are more accessible and acceptable to the members of their communities [4]. With worldwide recognition of the importance of CHWs in especially middle- and low-income countries, a shift towards the professionalisation of CHWs was inevitable. Ballard et al. contrast the differences between professional CHWs (‘proCHWs’) and CHWs, warning that the way in which the integration of CHWs into health systems happens can have a huge impact on the overall success of the health system [5].

**Figure 1:**
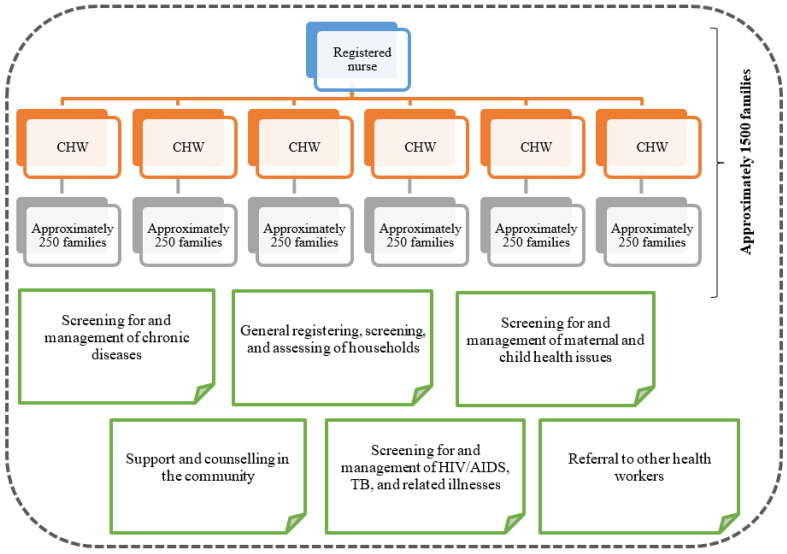
Structure of a municipal ward-based outreach team in the re-engineered PHC plan for SA.

The new PHC plan proposed for SA clearly provides for the inclusion of CHWs to assist with health care delivery in the community. CHWs are included as the key role-players in the municipal ward-based teams (see Figure 1), which are piloted in 11 districts across SA as of 2012. This forms part of the first phase of the implementation of the re-engineered PHC plan in districts with especially large numbers of underserved communities [6].

Previous studies have reported on challenges with the implementation of PHC programmes in countries where CHWs were key role-players [4,7,8]. With the pilot implementation of a similar PHC plan in South Africa since 2012, important questions have arisen: What are the main challenges being experienced by CHWs as key role-players in the pilot districts, particularly in the more rural areas? Are these challenges similar to or different from those identified in other countries? How can the major challenges possibly be addressed? In an attempt to address questions like these, the researchers regarded it as important to interact with the CHWs during the pilot phase of this national project. The study thus aimed to gain insider perspectives into the challenges CHWs experienced in a more rural NHI-pilot area of SA, and with these insights in mind, suggest recommendations that could strengthen the NHI plan moving forward in the roll-out thereof on a broader national level.

The article is structured to, firstly, give an overview of the roles and challenges of CHWs in NHI systems, integrated with South African perspectives on possible similar contexts. Secondly, the discussion focuses on the structure of the re-engineered South African PHC plan, also highlighting the similarities and differences with other PHC systems worldwide. The article concludes with practical suggestions for policy and practice.

### 1.1. Roles and Challenges of CHWs in the PHC Systems

The main aims on PHC systems worldwide, as in SA, are health promotion, the prevention of illness in communities and broadening access to the health care system [9,10,11,12,13,14]. CHWs are included in most of such decentralised, community-based systems as it could be more cost effective and promote self-reliance and local involvement [4]. CHWs could broaden the coverage of various services and improve service delivery and availability to poorer members of a community [4].

In an attempt to improve service delivery, health promotion and illness prevention in remote and rural communities, government-driven initiatives to restructure health care systems have been implemented by countries like Mexico, Brazil, Cuba, and China [1,9,12]. These initiatives included the implementation of neonatal screening, folic acid supplements during pregnancy, and training CHWs to assist in health promotion and illness prevention [9].

A move towards PHC systems is also found throughout the continent of Africa. The need to expand these PHC systems even more is, however, evident due to the growing burden of disease and small numbers of health workers in rural communities [14]. The government of Tanzania, for example, set out to expand health facilities to rural areas with the emphasis on the prevention of illness [11,12]. Much like in the case of SA, Tanzania faces challenges in terms of poverty, diminished physical and human resources, and a high population versus available health care staff ratio [11]. Due to the health care needs of a rapidly growing rural community, people were managed in their local village and then referred to specialised facilities if further management was required. This multi-tiered decentralised system is currently still operational in Tanzania [11].

Similarly, Mozambique expanded its PHC strategy in the 1980s by implementing a decentralization programme, moving health management from a provincial level to a district level. Improving integrated health care on a district level [13] has led to significantly improved health indicators [12], except for the Sofala Province in central Mozambique. The consistently high mortality rates in this area could be attributed to prevention strategies for HIV and tuberculosis (TB) not being optimal, as well as poor maternal and child health. The accomplishments in Mozambique, the culmination of improved community involvement, and the government’s vision to address the health requirements of the people in poorer communities are commendable [13].

The implementation of PHC in Uganda, Zambia, and Ghana resulted in a decreased infant and child mortality rate due to early screening and immunisation [12]. Community-based monitoring of public PHC providers in Uganda, in a randomised field experiment, concluded that encouraging community involvement in health care service led to improved service delivery by CHWs, which resulted in improved health outcomes, namely a decrease in child mortality rates and an increase in child weight [10]. Participation in health care implementation is positively influenced by community involvement, which leads to improved health outcomes.

SA faces similar health challenges as other African countries, with a large portion of the population living in poverty and in remote areas. Furthermore, SA faces growing HIV/AIDS and TB epidemics, an increase in violent crimes and accidents, as well as an increase in the risk factors for chronic diseases [15]. Vast numbers of people in SA are thus in desperate need of health care and assistance, much like the situation globally, as well as in the rest of Africa [3], increasing the demand on health care services. Against this background, the South African government aims to re-engineer the health care system, affording quality health care to the communities that are currently underserved and thus improving health outcomes in the country [2] through high-impact basic services at a low cost [4]. The ideal model of re-engineering health care in SA is thus one that focusses on the entire population, reaching communities, households and schools rather than individuals, with the primary aim of preventing illness and promoting health [16]. The heavy burden of HIV/AIDS and TB on the health care system of SA means a more cost-effective solution is welcomed within the South African context where service delivery and accessibility would be more readily available.

However, with the implementation of such an expanded, community-based PHC system, the management of key elements like technical support and supervision, transport, accountability, infrastructural support, and financing are all imperative to ensure success [4], and failure to address these factors will increase the risk of failure for the entire project [7]. Ahmed et al., in their recently conducted systematic review, confirmed the importance of not ‘seeing CHWs as a temporary sticking plaster’ if the impact of CHW programmes are to be optimised [8].

In South Africa’s redesigned PHC plan, the CHWs are expected to pursue the same goals as in other countries, namely promoting health and preventing disease within communities. When provided with proper training, oversight, and support, CHWs serve as a vital link between households and the broader healthcare system, helping to raise awareness of health-related topics [16]. In such a system, effective referrals and collaboration between CHWs and other healthcare professionals can strengthen efforts toward health promotion and disease prevention, ultimately supporting the government’s aim of building a high-quality NHI system. The structure in which this would take place is briefly discussed in the next section.

### 1.2. The South African Re-Engineered PHC Plan: Role of CHWs

Within the redesigned South African PHC plan, great emphasis is placed on health promotion and disease prevention when CHWs, as key role-players, screen and assess households with a specific focus on maternal and child health, HIV/AIDS, TB, and chronic diseases. CHWs are also expected to support and counsel members of the community and/or refer them to other health practitioners when necessary [17]. CHWs function within municipal ward-based outreach teams, which consist of a professional nurse and six CHWs (reporting directly to the nurse) (see Figure 1). Each community team takes responsibility for approximately 1500 families. The community population size thus informs the number of teams, and every CHW will be assigned around 250 families (see Figure 1).

Figure 1 illustrates the expected scope of activities of CHWs within communities, the complexity of their role when dealing with approximately 250 families, and the support structure based on one registered nurse for six CHWs. This structure suggests a possibility for challenges with the implementation of the NHI plan and was also alluded to in the previous literature (see Section 1.1). However, CHWs, as the key role-players, can provide valuable insider perspectives, specifically related to the key questions raised earlier, to detect any challenges (already in the pilot phase of implementation), and to propose recommendations and make possible changes to the South African NHI plan to enhance the roll-out of the plan on a broader national level. The methodology section provides information on the collection of data from the CHWs and other related aspects.

## 2. Materials and Methods

### 2.1. Study Design

A qualitative research approach utilising focus group interviews was employed in this study. The value of utilising a qualitative research approach was that it allowed for the collection of rich information from participants in the form of their perspectives as insiders. These perspectives were based on their own explanation of their world (as CHWs) and how they made sense of important events in their lives as CHWs [18]. This data allowed the researchers to explore and describe the uniqueness of the perspectives of CHWs in the South African context but also allowed for analysis in relation to perspectives from other countries.

### 2.2. Ethical Considerations

Permission to perform the study was obtained from the Faculty of Health Sciences Research Ethics Committee (HSREC) at the University of the Free State (ECUFS174/2013) and the Free State Department of Health. The study was conducted according to the Declaration of Helsinki. Written informed consent forms in Afrikaans, English, and/or Sesotho were signed by participants before commencement of the study. Additionally, confidentiality agreements were signed by the interpreter, research assistant, and participants agreed that discussions during the focus group interviews would be handled confidentially. Efforts to improve credibility, transferability, objectivity, and trustworthiness of data in this study included verbatim transcriptions of interviews, data coded by the researcher and an expert co-coder, and the use of a trained translator.

### 2.3. Study Setting

The study was conducted in the Thabo Mofutsanyane district in the Free State province, South Africa. This district is one of the ten Department of Health selected districts, and one of the more rural districts, to pilot the re-engineered PHC health plan. The Thabo Mofutsanyane district is situated closest to Bloemfontein, where the researcher resides, and was thus selected for inclusion based on convenience to accommodate costs and allow for time limitations.

### 2.4. Recruitment and Sampling Method

The researcher approached the registered nurse in the selected municipalities as a contact person to obtain the contact details of each of the CHWs. The researcher also posted advertisements in the municipality as well as in the municipal clinic to recruit all the CHWs in that area to take part in the study. The researcher, furthermore, attempted to contact the CHWs telephonically or via email, supplying all the necessary information regarding the study. Leaflets containing study information were left with the supervising nurse to distribute as she would have direct contact with all CHWs. One week before data collection, the researcher scheduled a meeting at each of the five clinics to recruit participants through an information session. Permission was obtained from the nurse to host these additional information sessions at the clinics during which the value and aims of the study were explained to CHWs to improve the response rate. No relationship existed between the researcher (facilitator) and CHWs before the study. All CHWs that arrived at the clinic on the day of data collection, as communicated during the recruitment process, were included in the study.

### 2.5. Study Population and Sample Size

Through purposive sampling, the study population included all the CHWs from the five municipalities (i.e., Setsoto, Dihlabeng, Nketoana, Maluti, and Phumelela) in the Thabo Mofutsanyane district, Free State, South Africa. Thirty-one CHWs were included in the study.

### 2.6. Inclusion and Exclusion Criteria

For inclusion in the study, participants had to be contracted as CHWs by the South African government for the pilot implementation of the re-engineered PHC plan and be able to understand Afrikaans, English, and/or Sesotho. Health care staff with formal training in nursing, medical, or any other allied health professions, as well as alternative healers in this community, were excluded from this study.

### 2.7. Pilot Study

A pilot study was performed in the Pixley ka Seme district (Northern Cape province) which is also included in the PHC plan pilot study. The entire procedure was tested exactly from start to finish. The information obtained through this pilot study was not included in the final data set as CHWs in this district are not part of the study population. The time needed to complete the data collection (demographic questionnaire and focus group interview) as well as the comprehensiveness and understandability of the questions was determined by the pilot study.

### 2.8. Data Collection

Data collection was done in a dedicated space (i.e., training room) at each of the five municipal clinics. The main data collection procedure consisted of five focus group interviews (one in each of the five municipalities). Based on the limited number of CHWs per municipality, multiple focus groups per municipality (to obtain data saturation) were not possible, but all CHWs were included in the study population to ensure optimal representation. Table 1 portrays the number of CHWs who participated per municipality.

Before the commencement of the focus group interviews, participants completed a survey on demographic aspects, such as the participants’ age, gender, and employment time as a CHW, merely to be able to describe the sample population.

To best accommodate participants, the demographic questionnaire and focus group interviews were available in the most common languages spoken in the Thabo Mofutsanyane district, namely Afrikaans, English, and Sesotho. The focus group interviews were conducted by the researcher in English, assisted by a translator (for Sesotho participants).

### 2.9. Trustworthiness and Rigor

In this study, the data collection tools and research procedure were tested during the pilot study, which contributed to reliability. Results from the pilot study and the main study could be compared to establish data consistency. The pilot study ensured the clarity and accuracy of questions.

The researcher ultimately aimed to obtain and report a truthful picture of what has been said and seen. Strategies contributing to achieving this included a pilot study to determine whether the group interview questions were accurate in obtaining the necessary information from participants. Participants were video- and audio-recorded by a video recorder positioned on a tripod, to ensure that the researcher could go back to the original data source for verification.

The following aspects were considered by the researcher to enhance the trustworthiness of this study:

- An in-depth description of the methodology before the focus group interviews as well as during reporting on the group interviews.
- Multiple data sources were used (field notes).
- An external person transcribed the data to avoid bias.
- A journal was kept (note keeping) to record the thought patterns of the researcher.
- The avoidance of generalisation by providing understanding from the perspective of CHWs in the specific district.
- Maintaining confidentiality.

### 2.10. Data Analysis

Both the researcher and co-coder independently organised the data in an Excel spreadsheet, according to the method described by Creswell, into categories, sub-categories, and themes [19]. In the final phase of data analysis, the researcher and co-coder compared their coding, discussed the coding, and where differences existed, adjusted upon agreement of changes. Member checking was not performed due to CHWs (participants) not having access to electronic devices (e.g., computers) and/or data to download/upload transcripts.

## 3. Results

The findings included in this section portray the insider perspectives of 31 participants in the five municipalities in the Thabo Mofutsanyane district, Free State, South Africa. Results are, thus, specifically linked to the South African context, but similarities with other parts of the world might be evident. Most participants were females (84%), with a mean age of 39 years. The CHWs’ work experience varied from two to three years and every participant had signed contracts of employment from the RSA DoH since 2011, when the re-engineered PHC plan was first introduced.

Four prominent themes (as major challenges) emerged during the data analysis process and include (1) the lack of support in conveying key health-related information to communities; (2) lack of interaction and communication with the Department of Health (DoH); (3) tensions within relationships; and (4) unattainable expectations built into the wide range of responsibilities. Where applicable, quotes are included that reflect how participants were emotionally affected by some of these challenges.

### 3.1. Lack of Support in Conveying Key Health Related Information to Communities

Health promotion is driven by information dissemination to community members, and was strongly emphasised as a key role in their duties by all participants in this study, and interestingly this point emerged very early in each of the five focus group interviews. With the quadruple burden of disease in South Africa, an important role reported on in our study by the CHWs is their (often unsupported) role of informing members of the community about the signs and symptoms of diseases like TB and HIV, how they are contracted and spread, and possible treatment options for these diseases.

*“We talk a lot to our patients about HIV and TB and we encourage them to go for regular testing to know their status in time”.*

(Participant 1, Municipality 5)

*“TB is very prevalent. Some people are informed, but I feel they are not informed enough. That is why we have health talks about how it is spread, how it is cured, that it is an infectious disease, but it can be cured and controlled”.*

(Participant 1, Municipality 1)

In addition to general education on illnesses like HIV and TB, community health programmes place strong emphasis on informing community members about topics such as family planning, contraception, and monitoring the health of infants and children. These focus areas are also among the key objectives of South Africa’s re-engineered PHC plan and were frequently mentioned by participants in this study. According to them, a significant portion of their work involves encouraging young pregnant women to breastfeed, by explaining the health and financial benefits of breastfeeding, as well as its role in fostering a strong mother-child bond. CHWs, furthermore, review clinic records of every child in the community to ensure immunisations and clinic visits are up to date. They educate mothers on the importance of the “Road to Health” booklets and stress the critical role vaccinations play in a child’s health.

### 3.2. Lack of Interaction and Communication with the Department of Health

Even though CHWs seemingly perform a commendable job on health promotion and the prevention of illness in their communities (as alluded to above), participants mentioned that no representatives from the Department of Health (DoH) have interacted with them since 2011 when they were initially contracted. CHWs feel they were left to fend for themselves, must cope by themselves, and were forgotten about.

*“Nobody asks us “What are you doing,” or “What are your challenges?”*

(Participant 4, Municipality 5)

*“Nobody ever takes us seriously”.*

(Participant 4, Municipality 1)

*“We struggle without a team leader. It has been more than three years now and we are still being side-stepped. Nobody tells us what’s going on”.*

(Participant 3, Municipality 2)

If regular interaction with the DoH representatives was in place in the NHI pilot district included in this study, the physical needs expressed by CHWs, namely shoes (many CHWs have to walk far to the homes of patients), stationary, name badges (for credibility and trust), uniforms, transport, umbrellas (for heat and rain), and bags in which to carry their files, could also have been addressed.

*“We will look much [sic] more presentable to the community if we have these things”.*

(Participant 2, Municipality 5)

These requests seem insignificant to a person not familiar with the scope of the CHWs’ everyday tasks, yet these issues have never been voiced or addressed, due to the lack of support for the CHWs.

### 3.3. Tension Within Different Relationships

CHWs included in this study referred to tensions in different relationships, such as relationships with clinic sisters (i.e., registered nurses), community members, and their own families.

*“The sister will cut you off if you are trying to say something. Later you start to lose interest in your work. They take us as lower persons. The sister thinks we want to know more than her and then she gets very angry. Then we don’t show our skills anymore”.*

(Participant 3, Municipality 4)

When describing their perspectives regarding their relationship with the community, study participants often described their perspectives within the broader context of ‘harm’. Participants alluded to dog attacks (or people putting their dogs on them), rape and assault, general abuse by certain patients, and their fear of mentally ill patients in the community.

*“We constantly feel threatened. What will become of us if we are injured?”*

(Participant 8, Municipality 3)

*“We try our best, but we get criticized all the time. People put their dogs on us and expect food, medication, and money from us”.*

(Participant 3, Municipality 5)

Another tension within the relationship between the CHWs and the community was due to referrals of community members to health care facilities (mainly clinics). CHWs reported visiting the homes of the community members to inform them about the various services that are available at the local clinic. Even though referrals of community members are seen as one of their core functions, CHWs in this study were disappointed when performing this important function. Their disappointment was due to the poor level of service the community members received at the clinics, which ultimately reflected poorly on them as CHWs, as well as negatively influenced their credibility amongst community members.

*“We refer our patients to the clinics but there is no help for them there”.*

(Participant 6, Municipality 5)

*“The clerks don’t understand the referral process, so our patients don’t get seen when we refer them”.*

(Participant 4, Municipality 4)

Furthermore, to improve service delivery to the mass of the community––a major objective of the re-engineered PHC plan for SA––the CHWs in this study focused strongly on the dispensation and/or monitoring of adherence to medication. CHWs in this study reported on many patients discontinuing the use of their medication to deliberately avoid the long queues, extensive waiting times, or unavailability of medication at the clinics. Other patients do not have transport (or money for transport) to get to the clinics and subsequently discontinue the use of their medication. CHWs, however, continue to identify and document these issues and encourage community members to resume their treatment despite the difficulties mentioned above. CHWs in this study also made suggestions of how they feel they could alleviate a lot of these challenges themselves, by for example taking the community members’ medication to their homes.

*“We are able to take the clinic to the people”.*

(Participant 4, Municipality 3)

### 3.4. Unattainable Expectations Built into the Wide Range of Responsibilities

Another challenge for CHWs within the re-engineered PHC plan is the lack of insight and/or transparency regarding their responsibilities. This could possibly also be attributed to poor communication, teamwork, and relationships between CHWs and registered nurses at clinics, as previously discussed in this article.

CHWs were trained to take the vital signs of patients as part of their initial training programme, but some participants felt unsure of their exact responsibility regarding this task, as they are not permitted to do this in the homes of the people yet expected to take vital signs readings in the clinics.

*“They make us do it at the clinics, but we are not permitted to do it in the homes”.*

(Participant 3, Municipality 3)

Another interesting note from this quote is the discrepancy between CHWs’ responsibilities when working in the community itself and when in the clinic (i.e., taking vital signs and dispensing medication). CHWs in this study clearly voiced their frustration in terms of the existing discrepancies between what they were trained to do and what they ended up doing all day. They were well-trained in HIV screening and ante- and post-natal care, yet they are kept busy for the biggest part of their day doing registrations of all the households.

Even though CHWs felt confident in certain areas, they still stated that their training was too short and too long ago (upon their appointment as CHWs) and that as they encounter real-life situations, they require continued education. One area of uncertainty was identified as the education of families and caretakers of patients with illnesses and/or weaknesses as they observed that most families did not have an idea of how to manage their patients at home.

After critical reflection on these insider perspectives from CHWs, the authors make some recommendations for policy and practice, in the next section. If implemented, these recommendations could enhance CHWs’ effectiveness and/or contribution they could make to the further roll-out of the NHI in South Africa, contributing positively to the health of all communities.

## 4. Discussion and Recommendations from Study Findings for Policy and Practice

The results guided the discussion and informed the recommendations for policy and practice. The authors suggest that consideration be given to four specific recommendations, which could address several challenges highlighted by the CHWs included in this study. If not addressed, the danger exists that these perspectives/feelings of CHWs may in future cause these eager workers to abandon their calling and give up. This is a scenario a country like SA cannot afford with its growing burden of disease, financial constraints, and existing shortage of health workers to serve the masses.

The four recommendations from this study are the (1) appointment of a dedicated CHW team leader; (2) appointment of a dedicated CHW registered nurse; (3) continued education and training for CHWs with an accompanying portfolio of evidence; and (4) hosting of regular awareness campaigns.

### 4.1. Appointment of a Dedicated CHW Team Leader

Although a support structure has been proposed for the South African re-engineered PHC plan, it seems to not be functioning optimally. The challenge might be due to a registered nurse being the team leader and the only support structure for CHWs within the system (see Figure 1). Schneider et al. previously alluded to the tension between CHWs and registered nurses when the role of CHWs were investigated in the Free State province [16]. These nurses were extremely reluctant for CHWs to be issued uniforms, made them feel unwelcome and as if they did not belong in the community. The feelings of being rejected and taken for granted were documented by CHWs during Schneider et al.’s investigation and when asked why they did not leave their jobs they responded with the argument that they love what they do and keep hoping things would change [16]. It is a matter of concern that although this challenge was already known to the DoH when planning the re-engineered PHC plan for SA, no changes were made to the structure of functioning, and similar results were found almost 10 years later in our study.

Due to the tensions within the relationship of CHWs with the registered nurses, appointing a dedicated CHW team leader within each municipality may alleviate these tensions and create a more positive support structure. The lack of support to CHWs in our study is an important challenge to note, as the literature states that barriers such as a lack of support and/or structure, poor task allocation, and poor supervision contribute to a high attrition rate amongst CHWs [20]. This has even been previously confirmed in a South African study by Lehmann et al. clearly stating that CHWs have a great service to offer the community by implementing certain health care principles that would impact the health outcomes in SA tremendously, but that these CHWs require continuous support [4]. Authors reporting in a recent study from Indonesia confirmed the importance of a detailed supervision protocol for CHWs, to ensure effectiveness of such a system [20]. Therefore, supervision and a strong support system are two non-negotiable requirements for a successful health outcome.

**Figure 2:**
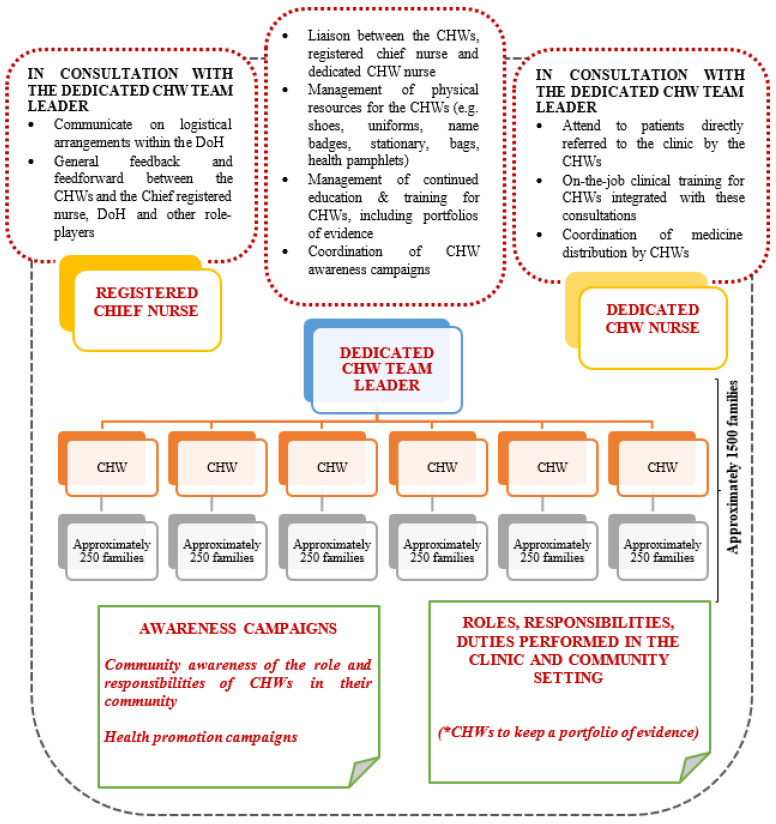
Proposed adapted structure of a municipal ward-based outreach team in the re-engineered PHC plan for SA.

Regarding our recommendations for the appointment of a CHW team leader, such a CHW team leader could act as the liaison between the CHWs and the rest of the PHC team and could fulfil coordination functions, amongst others, linked to ordering and provisioning of physical resources needed by CHWs to fulfil their duties, improved service delivery, improved communication and the facilitation of continuous development and training of CHWs (see Figure 2).

Training was an important aspect alluded to by the CHWs throughout the focus group interviews and could become an important focus in the responsibilities of the dedicated CHW team leader. Different training programmes have previously been implemented for CHWs in the different South African provinces, with no evidence of a standardised training curriculum. This resulted in different emphasis placed upon certain aspects of health care in different communities [21]. Lehmann et al. also state that very limited information about the precise detail of CHW training in SA exists and that the literature only briefly mentions the training provided to CHWs in other African countries, such as Nigeria, Somalia, and Tanzania [4]. Regarding the training received by the participants in our study, two areas were highlighted as having received great attention, namely mother and child health as well as HIV management. Evidence of continued training in areas where CHWs felt less competent could become part of the portfolio of evidence (as previously mentioned) and be coordinated by the dedicated CHW team leader (see Figure 2).

### 4.2. Appointment of a Dedicated CHW Registered Nurse

To address the reported challenge regarding referrals and poor service delivery at clinics when referred, the authors suggest that an operational procedure be developed for service delivery to patients referred to clinics by CHWs. The South African DoH in 2009 acknowledged the problems within the PHC referral system. Referrals from CHWs to other health care professionals were introduced in the NHI plan as an option to address the challenges [22]; however, from the findings in this study it seems to remain a challenge. As CHWs would have already completed the screening of these patients, the service could be much more specific and streamlined within the operational functions of any clinic, e.g., through a dedicated CHW nurse for these patients (see Figure 2).

Such a specific operational system (as in Figure 2) could also address the challenge related to the apparent lack of insight or transparency in the responsibilities of CHWs. If CHWs refer only to a specific CHW nurse, they could build better teamwork relations, improve communication and enhance service delivery to communities. It could also relieve some of the tension in the current relationship between CHWs and registered nurses at clinics. If the relationship between the CHWs and the proposed, dedicated CHW registered nurse is further enhanced and all parties are clear on the skills and responsibilities of all within the PHC team, additional responsibilities could be safely introduced, e.g., the distribution of medication. Such a function could be coordinated between the suggested dedicated CHW team leader and dedicated CHW registered nurse and become an important mechanism in addressing the TB as well as HIV/AIDS challenges faced by SA.

Such a solution is supported by previous studies, e.g., a study performed in 75 villages in Western Kenya who recruited 75 CHWs from those areas and trained them to become the key driving force of medication for Schistosomiasis (Bilharzia) [23]. Kenya experienced great challenges with the control of this particular illness because, although medication was available, it was being stored at local facilities (clinics) and not accessible by the people most in need of treatment. Through this initiative, the most patients ever recorded received medication and an overall decline in reported infections was noted. Kenya is currently developing countrywide programmes that mobilise CHWs to participate as mass drug distributers to the poor rural communities, as their study indicated that CHWs have a much larger coverage and can reach the masses that are in dire need of medication [23].

### 4.3. Continued Education and Training of CHWs, with an Accompanying Portfolio of Evidence

To address the challenge with regard to lack of insight into the skills of CHWs when introduced in the community, CHWs in this study suggested that they receive an official certificate related to all the training they attended and/or skills they mastered as evidence of their competencies. The authors, in addition, suggest that CHWs are expected to keep an up-to-date portfolio of evidence on all duties they perform in both the community setting as well as the clinic setting (see Figure 2).

### 4.4. Awareness Campaigns

The CHWs in this study expressed their concern regarding the lack of awareness of communities regarding their roles and responsibilities. The authors, therefore, suggest awareness campaigns to be run in communities under the leadership of the dedicated CHW team leader (see Figure 2) to alert communities to the valuable role CHWs play in the promotion of health and prevention of illness within their community. As demotivation seems to be a common manifestation among CHWs in our study, mostly due to tension within their relationships, it is important to take note of the study of Brunie et al. [24] in Uganda who found that public acknowledgement and appreciation improve the motivation of CHWs to keep doing their best. LeBan et al. reported on CHWs’ ‘intermediary position between the health system and the community’. Inherently, such a position will be associated with tensions due to differences in needs and expectations [25] but also hold immense potential to bring these stakeholders closer together if managed correctly. These authors propose a multi-stakeholder approach, including leaders from national to local health sectors, local government representatives, and representatives from community organisations. Currently, the re-engineered South African PHC system does not pertinently make provision for such interactions, which could explain some of the feelings of demotivation expressed by participants.

Awareness campaigns could furthermore focus on specific health promotion topics, as included in the roles and responsibilities of CHWs. In our study, CHWs referred to the health information they give to community members especially with regard to TB and/or HIV. This finding is in line with the available literature, where Thomas et al. [26] refer to community education as one of the central roles of any CHW. A study on the role of CHWs performed in Northeast Brazil also showed that these workers all spend a great amount of time on conveying information regarding health promotion and prevention of illness [27]. In the USA, the role of CHWs is not only providing health education and information, but also on how the community can access health services [28].

However, the implementation of such an adapted structure (see Figure 2) in South Africa would require additional funding. Perry et al. allude to the ‘need for a marked increase in sustainable funding for CHW programmes to reap the benefits such a system could hold for any country’ [29]. The South African government has shown their commitment to improved health care through a CHW programme, but additional funding will be essential to address the gaps identified in this study.

## 5. Conclusions

The overwhelming feeling amongst CHWs in the focus groups was that of discouragement, and they voiced many contributing factors leading them to feel hopeless while fulfilling their role in the community. However, this study effectively identified the four major challenges contributing to the CHWs perspectives, and similarities and/or differences with other CHWs across the world were highlighted. The findings should, still, only be interpreted against the limitations of the study, namely that the research was conducted in one district in the Free State, and the findings can therefore not be generalised to represent the perceptions, attitudes, and practices of all CHWs forming part of the NHI-pilot implementation in South Africa.

Further value from this study might be in the focused recommendations for policy and practice proposed in this article (see Section 4), as it could serve as a basis for further research. Similar studies could be conducted in all ten NHI-pilot districts to compare findings and draw conclusions from similarities and differences between the districts (also alluding to rural and urban differences), or further research could focus on the recommendations from this study and the possible effects they could have on the perspectives (and effectiveness) of CHWs within communities.

## Figures and Tables

**Table 1:** Number of participants per focus group (per municipality).

Municipality	Number of CHWs (*n* = 31)
Setsoto	11
Dihlabeng	5
Nketoana	6
Maluti	6
Phumelela	3

## Data Availability

The data presented in this study are available on request from the corresponding author due to privacy. The data will only be stored for a limited period of time, as required by law.

## Abbreviations

The following abbreviations are used in this manuscript:

NHI	National Health Insurance
PHC	Primary Health Care
CHWs	Community Health Workers
SA	South Africa
HIV	Human Immunodeficiency Virus
TB	Tuberculosis

## References

[1] Wu Y., Zhang Z., Zhao N., Yan Y., Zhao L., Song Q., Ma R., Li C., Li J., Liu S. (2022). Primary health care in China: A decade of development after the 2009 health care reform. Health Care Sci..

[2] Republic of South Africa, Department of Health (RSA DoH) (2011). National Health Insurance in South Africa.

[3] Mupara L.M., Mogaka J.J., Brieger W.R., Tsoka-Gwegweni J.M. (2023). Community Health Worker programmes’ integration into national health systems: Scoping review. Afr. J. Prm Health Care Fam. Med..

[4] Lehmann U., Friedman I., Sanders D. (2004). Review of the Utilization and Effectiveness of Community-Based Health Workers in Africa.

[5] Ballard M., Dahn B., O’Donovan J., Jiménez A., Kawooya P., Raghavan M., Ganjian N., Johnson A.D., Boxer C., Gray K. (2025). One term to transform: Universal health coverage through professional community health workers. Lancet.

[6] Republic of South Africa, Department of Health (RSA DoH) (2012). Provincial Guidelines for the Implementation of the Three Streams of PHC Re-Engineering.

[7] Nxumalo N., Gouge J., Thomas L. (2013). Outreach services to improve access to health care in SA: Lessons from three community health worker programmes. Glob. Health Action..

[8] Ahmed S., Chase L.E., Wagnild J., Akhter N., Sturridge S., Clarke A., Chowdhary P., Mukami D., Kasim A., Hampshire K. (2022). Community health workers and health equity in low- and middle-income countries: Systematic review and recommendations for policy and practice. Int. J. Equity Health.

[9] Sepúlveda J., Bustreo F., Tapia R., Rivera J., Lozano R., Oláiz G., Partida V., García-García L., Valdespino J.L. (2006). Improvement of child survival in Mexico: The diagonal approach. Lancet.

[10] Bjorkman M., Svensson J. (2009). Power to the people: Evidence from a randomized field experiment on community-based monitory in Uganda. Q. J. Econ..

[11] Kwesigabo G., Mwangu M.A., Kakoko D.C., Warriner I., Mkony C.A., Killewo J., Macfarlane S.B., Kaaya E.E., Freeman P. (2012). Tanzania’s health system and workforce crisis. J. Public Health Policy.

[12] Magawa R. Primary Health Care Implementation: A Brief Review. https://www.polity.org.za/article/primary-health-care-implementation-a-brief-review-2012-08-21.

[13] Sherr K., Cuembelo F., Michel C., Gimbel S., Micek M., Kariaganis M., Pio A., Manuel J.L., Pfeiffer J., Gloyd S. (2013). Strengthening integrated primary health care in Sofala, Mozambique. BMC Health Serv. Res..

[14] Ayankogbe O.O. (2014). Building capacity for African Primary Care Research. Afr. J. Prm. Health Care Fam. Med..

[15] Mayosi B.M., Lawn J.E., Van Niekerk A., Bradshaw D., Abdool-Karim S.S., Coovadia H.M. (2012). Health in South Africa: Changes and challenges since 2009. Lancet.

[16] Schneider H. (2012). M&E of PHC Outreach Teams. National Health Assembly. https://www.hst.org.za/publications/South%20African%20Health%20Reviews/Chap%207%20WBOTS.pdf.

[17] Okafor U.B., Obasanjo I., Ter Goon D. (2024). Important but Neglected: Job description of community health workers in the Eastern Cape: A qualitative study. Open Public Health J..

[18] McMillan J.H., Schumacher S. (2014). Research in Education: Evidence-Based Inquiry.

[19] Creswell J.W. (2014). Research Design. Qualitative, Quantitative, and Mixed Methods Approaches.

[20] Nida S., Tyas A.S.A., Putri N.E., Larasanti A., Widoyopi A.A., Simayyah R., Listiana S., Espressivo A. (2024). A systematic review of the types, workload, and supervision mechanism of community health workers: Lessons learned for Indonesia. BMC Prim. Care.

[21] Languza N., Lushaba T., Magingxa N., Masuku M., Ngubo T. (2011). Community Health Workers: A Brief Description of the HST Experience. Health Systems Trust. https://www.hst.org.za/publications/HST%20Publications/CHWs_HSTexp022011.pdf.

[22] Republic of South Africa, Department of Health (RSA DoH) (2009). National Health Insurance in South Africa.

[23] Omedo M.O., Matey E.J., Awiti A., Ogutu M., Alaii J., Karanja D.M.S., Montgomery S.P., Secor W.E., Mwinzi P.N.M. (2012). Community health workers’ experiences and perspectives on mass drug administration for Schistosomiasis control in Western Kenya: The SCORE project. Am. J. Trop. Med. Hyg..

[24] Brunie A., Wamala-Mucheri P., Otterness C., Akol A., Chen M., Bufumbo L., Weaver M. (2014). Keeping community health workers in Uganda motivated: Key challenges, facilitators, and preferred program inputs. Glob. Health Sci. Pract..

[25] LeBan K., Kok M., Perry H.B. (2021). Community health workers at the dawn of a new era: 9. CHWs’ relationships with the health system and communities. Health Res. Policy Syst..

[26] Thomas L.S., Pillay Y., Buch E. (2024). Community perceptions of community health worker effectiveness: Contributions to health behaviour change in an urban health district in South Africa. SAMJ.

[27] Grossman-Kahn R., Schoen J., Mallett J.W., Brentani A., Kaselitz E., Heisler M. (2018). Challenges facing community health workers in Brazil’s family health strategy: A qualitative study. Int. J. Health Plan. Manag..

[28] O’Brien M.J., Squires A.P., Bixby R.A., Larson S.C. (2009). Role development of community health workers. Am. J. Prev. Med..

[29] Perry H.B., Chowdhury M., Were M., LeBan K., Crigler L., Lewin S., Musoke D., Kok M., Scott K., Ballard M. (2021). Community health workers at the dawn of a new era: 11. CHWs leading the way to “Health for All”. Health Res. Policy Syst..

